# Comparison Between Single-Use Flexible Ureteroscope and Reusable Flexible Ureteroscope for Upper Urinary Calculi: A Systematic Review and Meta-Analysis

**DOI:** 10.3389/fsurg.2021.691170

**Published:** 2021-10-13

**Authors:** Chunyang Meng, Lei Peng, Jinze Li, Yunxiang Li, Jinming Li, Ji Wu

**Affiliations:** ^1^Department of Urology, Nanchong Central Hospital, The Second Clinical Medical College, North Sichuan Medical College (University), Nanchong, China; ^2^West China School of Medicine, Sichuan University, Chengdu, China; ^3^Department of Urology, The Affiliated Hospital of Medical College, North Sichuan Medical College (University), Nanchong, China

**Keywords:** upper urinary calculi, flexible ureteroscope, single-use, reusable, meta-analysis

## Abstract

**Objective:** This article explores the differences in the effectiveness and safety of the treatment of the upper urinary calculi between single-use flexible ureteroscope (su-fURS) and reusable flexible ureteroscope (ru-fURS).

**Methods:** We systematically searched PubMed, Embase, Cochrane Library, Scopus database, and CNKI databases within a period from the date of database establishment to November 2020. Stata 16 was used for calculation and statistical analyses.

**Results:** A total of 1,020 patients were included in the seven studies. The statistical differences were only found in the Clavien–Dindo grade II postoperative complication [odds ratio (OR) 0.47; 95% CI 0.23–0.98; *p* = 0.04]. No significant statistical differences were observed in operative time (OT), estimated blood loss (EBL), length of hospital stay (LOS), and stone-free rate (SFR).

**Conclusion:** Our meta-analysis results demonstrate that su-fURS, compared with ru-fURS, has similar effectiveness and better security for treating upper urinary calculi.

## Introduction

Urolithiasis is one of the most common diseases in urology, which has a high incidence in the world. Its incidence rate varies, among which North America has the highest incidence rate of 7–13%, Europe has the highest incidence rate of 5–9%, and Asia has a relatively low incidence rate of 1–5% (1). Kidney stones can lead to renal colic, urinary tract infection, and obstruction and are also risk factors for chronic kidney disease (2). The treatment of upper urinary calculi has always been the focal point of medical research. Surgical treatment was the main treatment method of the upper urinary tract stone. Open surgery was highly traumatic and could only be used for some special patients. Percutaneous nephrolithotomy has the highest rate of surgical exclusion for large stones and multiple kidney stones. Tubeless minimally invasive percutaneous nephrolithotomy may be a probable choice for strictly chosen patients (3). The flexible ureteroscopes (f-URSs) were taking an essential role in recent years and the new thulium laser system during ureteroscope was giving interesting results (4).

In the recent revision of the European guidelines on the management of urolithiasis, ureteroscope was recommended as a first-line management option, especially for stones measuring between 10 and 20 mm. Moreover, for stones > 1.5 cm in the lower pole, a flexible ureteroscope is also one of the recommendations (5).

However, the existing limitations on reusable flexible ureteroscope (ru-fURS) include a high initial purchase cost, high expenditures for repair, and a risk of cross-infection (6). Studies have shown that even when ru-fURS was cleaned manually and disinfected by hydrogen peroxide gas, contamination could still be found (7, 8). For solving these problems with existing ru-fURS, a single-use flexible ureteroscope (su-fURS) has been proposed and has recently come to gain achievements (9, 10).

In fact, for su-fURS, there is still a lack of high-level evidence to compare its safety and efficiency with that of ru-fURS. Therefore, the purpose of this meta-analysis is to compare the clinical efficacy and safety of the treatment of the patients with upper urinary calculi between the two types of scopes.

## Methods

### Literature Search and Eligible Criteria

A systematic search in PubMed, Embase, Cochrane Library, Scopus database, and CNKI databases was performed to identify the studies published from the date of database establishment to November 2020. Search terms included: “ureteroscope,” “flexible ureteroscope,” “single-use,” “disposable,” “reusable,” “upper urinary calculi,” “kidney stone,” “ureteral calculi,” and the search was not restricted by language. Besides, manual retrieval from the references of subject-related studies was performed to broaden the search.

Studies meeting the following inclusion criteria were listed as follows: (1) patients diagnosed as upper urinary calculi by a urologist; (2) comparison of su-fURS with ru-fURS; (3) any size of the stones and a similar number of surgeries; (4) full papers containing at least one outcome parameters such as operative time (OT), estimated blood loss (EBL), length of hospital stay (LOS), stone-free rate (SFR), and complications; and (5) the type of articles should be a prospective controlled study, cohort study, retrospective study, or randomized controlled study. Duplicate studies, reviews, case reports, letters, irrelevant studies about our topic, and studies from which available data could not be extracted were excluded. OT, SFR, and complications were the primary outcomes. The secondary outcomes were EBL and LOS.

This process was independently performed by the two authors (JZL and LP) and the differences between the authors were settled by consultation. The third reviewer (YXL) was involved in the judgment if an agreement could not be reached.

### Data Extraction

We extracted the following data from each study into the meta-analysis: author, publication year, study design, sample size, detailed information of ureteroscopes, OT, EBL, LOS, SFR, and complications. When continuous variables were reported as median and range in the main literature, we calculated the mean and SD (11).

### Study Quality Assessment

Based on the results available, we used the Jadad scale (12) to assess the randomized controlled trials (RCTs) and the Newcastle–Ottawa Scale (NOS) scoring rules (13) for non-randomized controlled studies. The Jadad score ranges from zero to seven points. A score lower than four should be considered to indicate a low-quality study; else, it should be considered a high-quality study. The NOS scale is a total of nine stars and more than six stars should be considered as high-quality research.

### Risk of Bias Assessments

Not only using ROBINS-I tool for non-randomized studies but also using ROB2 for RCTs to evaluate a risk of bias. The ROBINS-I tool included seven domains: confounding bias, selection bias, bias in measurement classification of interventions, bias due to deviations from intended interventions, bias due to missing data, bias in the measurement of outcomes, and bias in the selection of the reported result (14). The ROB2 tool contained a randomization process, deviations from intended interventions, missing outcome data, measurement of the outcome, and selection of the reported result (15).

### Statistical Analysis

This meta-analysis was performed by using Stata 16 for the statistical analysis. The odds ratio (OR) and mean difference (MD) were used to evaluate the dichotomous and continuous data, respectively, and a 95% CI and *p*-value were calculated. *p* < 0.05 was considered as statistically significant. *I*-square tests were used to verify the heterogeneity between the included studies. Meanwhile, we performed a sensitivity analysis to interpret the potential source of heterogeneity, if the heterogeneity is more than 50%.

### Registration

This study registered on the PROSPERO and the registration number was CRD42021230884.

## Results

### Description of Studies

**Figure 1:**
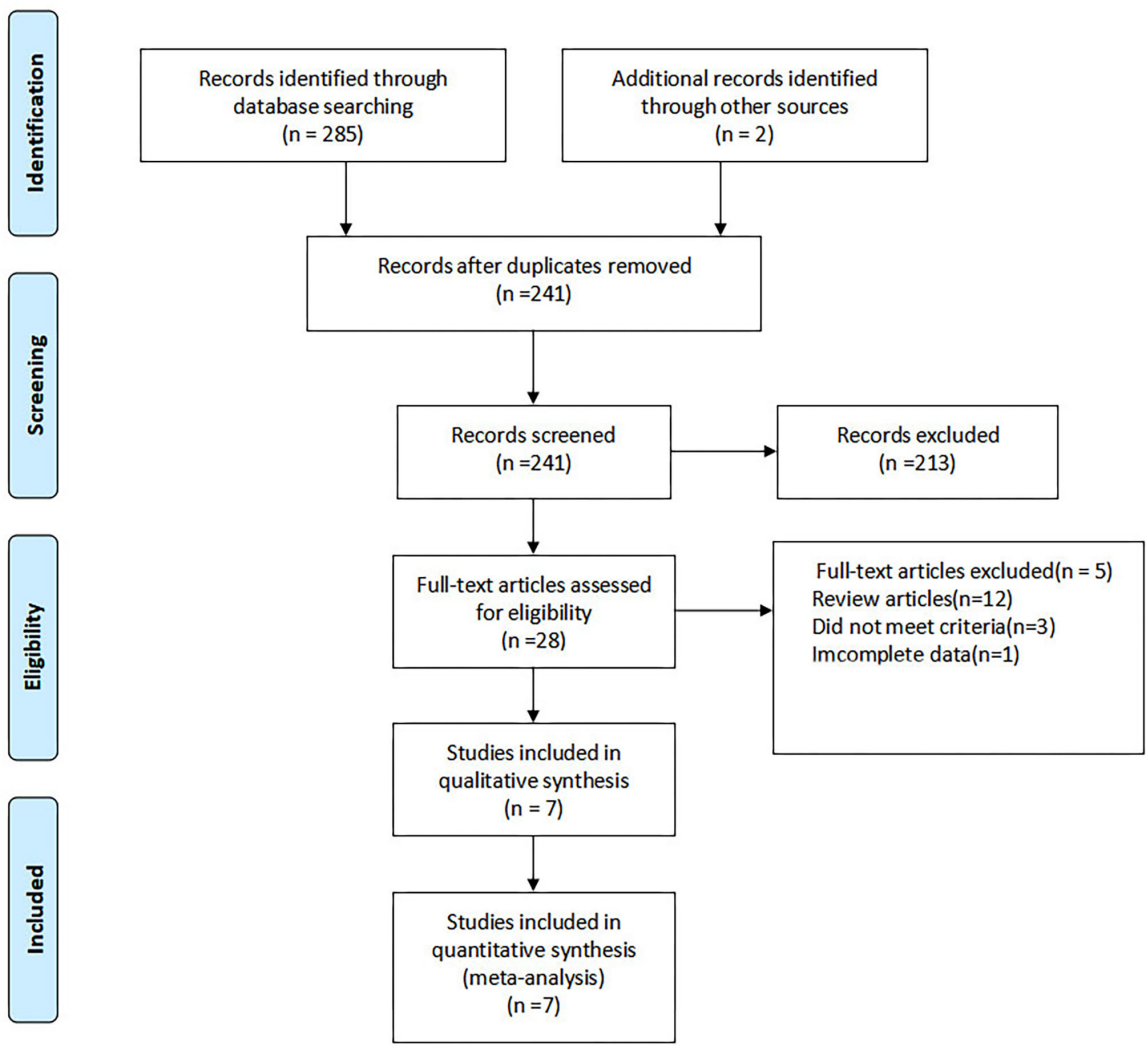
Flow diagram of studies selection process.

A total of 287 studies were identified, out of which 28 studies were full-text reviewed and seven studies were eventually selected (16–22). The screening process is shown in Figure 1. Table 1 lists the characteristics of the included studies. The seven included studies were published between 2015 and 2020 and a total of 1,020 patients and the sample size of the studies ranged from 61 to 360. Among them, four studies had a prospective design and three studies had the RCTs.

**Table 1:** Baseline characteristic of included studies.

**Studies, year**	**Country**	**Intervention su-fURS/ru-fURS**	**No.of patients**	**Age (year)**	**Number of stones**	**Stone size (mm)**	**Study design**	**Quality score**
Zhu (2020)	China	PU3022A Flex-X2	45 45	45.1 ± 9.3 44.5 ± 8.5	NA	11.6 ± 5.0 8.7 ± 3.0	RCT	7^a^
Qi (2019)	China	ZebraScope URF-V	63 63	51.84 ± 13.16 53.25 ± 12.11	1.17 ± 0.92 1.95 ± 1.02	NA NA	RCT	6^a^
Mager (2018)	Germany	Lithovue Flex-X2S, Flex-XC	60 62	54 ± 17 59 ± 16	NA	NA NA	prospective	8^b^
Kam (2019)	Australia	Lithovue URF-V2	55 64	53.5 (46.2–60.7)^c^ 53.3 (47.6–59.0)^c^	2.3 (1.6–2.9)^c^ 2.0 (1.7–2.4)^c^	14.7 (11.2–18.1)^c^ 13.3 (11.0–15.6)^c^	prospective	7^b^
Usawachintachit (2017)	U.S.A	Lithovue URF-P6	92 50	55.8 ± 15.1 50.5 ± 12.6	2.0 ± 1.7 1.6 ± 1.3	14.7 ± 9.9 16.3 ± 12.2	prospective	8^b^
Ding (2015)	China	PolyScope URF-P5	180 180	50.5 ± 12.8 51.1 ± 13.7	1.53 ± 0.7 1.58 ± 0.94	NA NA	RCT	6^a^
Salvado (2019)	Chile	Uscope3022 Cobra	31 30	50.4 ± 13.8 49.9 ± 16.5	NA	10.8 ± 5.0 9.0 ± 3.3	prospective	7^b^

*su-fURS, single-use flexible ureteroscope; ru-fURS, reusable flexible ureteroscope; NA, not available; ±, refers to standard deviation*.

a *using Jadad scale;*

b *using NOS scoring rule;*

c *mean (95%CI)*.

### Quality Assessment

Based on the Jadad scale and the NOS scoring rules, we have listed the final study quality scores in Table 1.

### Risk of Bias of Included RCTs

The ROB2 tool was performed to evaluate the risk of bias and the major weakness was in the domains of deviations from intended interventions. The final results were upload to Supplementary Materials.

### Risk of Bias of Included Non-randomized Studies

The ROBINS-I tool was used to assess the risk of bias and the main weakness was in the selection bias. The final results suggested that all the comparative studies had a moderate risk of bias.

## Perioperative Outcomes

### Operative Time

**Figure 2:**
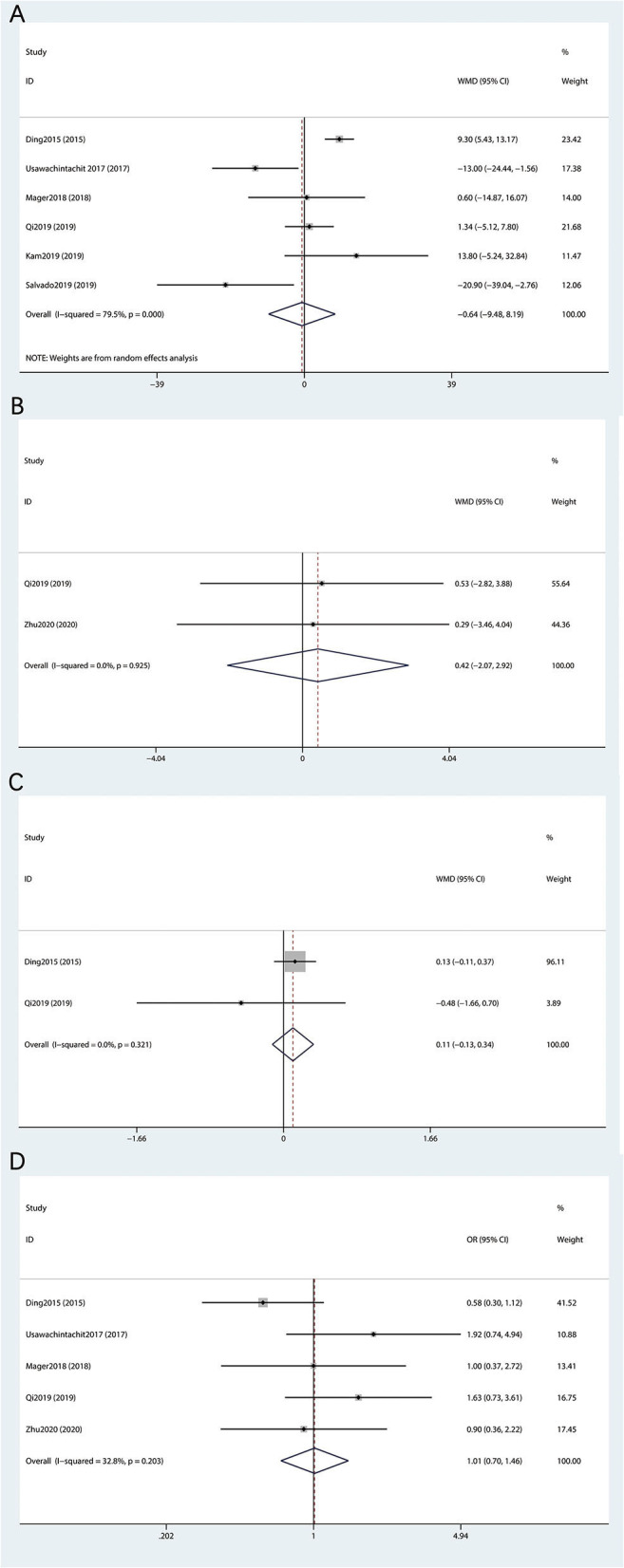
Forest plot of perioperative outcomes between the su-fURS and ru-fURS. **(A)** operative time, **(B)** estimated blood loss, **(C)** length of hospital stay, and **(D)** stone-free rate.

Data of OT were reported in six studies (16–21) including 930 patients. The heterogeneity test results suggested that the heterogeneity among studies is high (*I*^2^ > 50%) and a random effects model was used. The final meta-analysis showed no statistical difference between the su-fURS and ru-fURS (MD: 0.64; 95% CI 9.48–8.19; *p* = 0.886; Figure 2A).

### Estimated Blood Loss

A total of two studies related to EBL (19, 22) after the operation, including 1,287 patients, and a fixed effects model was used according to the results of heterogeneity analysis (*I*^2^ = 0%). The last result showed that the difference was not statistically significant between the su-fURS and ru-fURS (MD: 0.42; 95% CI 2.07–2.92; *p* = 0.74; Figure 2B).

## Prognostic Outcomes

### Length of Hospital Stay

Among the two studies (16, 19) on the LOS, there was no obvious heterogeneity and we used a fixed effects model for meta-analysis. The final outcomes indicated the absence of statistically significant difference between the su-fURS and ru-fURS (MD: 0.11; 95% CI 0.13–0.34; *p* = 0.371; Figure 2C).

### Stone-Free Rate

The SFR was recorded in five out of seven studies (16, 18, 19, 21, 22) containing 840 patients. Since there was no outcome of the heterogeneity test (*I*^2^ = 32.8%), a fixed effects model was used. No statistical differences were observed between the su-fURS and ru-fURS (MD: 1.01; 95% CI 0.70–1.46; *p* = 0.948; Figure 2D).

### Complications

We performed a meta-analysis of the complication after surgery and based on the heterogeneity test, a fixed effects model was used. The final results indicated that there was no statistically significant difference between the su-fURS and ru-fURS (OR 0.93; 95% CI 0.66–1.29; *p* = 0.646; Table 2).

**Table 2:** The meta-analysis of postoperative complication.

**complication**	**No. of studies**	**No. of patients**	**OR (95% CI)**	***P*** **-value**	**Heterogeneity (I^2^)**
		**su-fURS /ru-fURS**			
Clavien–Dindo grade I	7	526/494	1.05 (0.72, 1.55)	0.79	1.7%
Clavien–Dindo grade II	5	315/284	0.47 (0.23, 0.98)	0.04	0%
Clavien–Dindo grade III–V	4	395/355	1.11 (0.52, 2.36)	0.79	0%
Total	7	526/494	0.93 (0.66, 1.29)	0.65	41.7%

*OR, odds ratios; CI, confidence interval; su-fURS, single-use flexible ureteroscope; ru-fURS, reusable flexible ureteroscope*.

Furthermore, based on the Clavien–Dindo grades for the postoperative complications, we also performed subgroup analysis. The results of the subgroup analysis interpreted that significant differences were only observed in the Clavien–Dindo grade II postoperative complication: Clavien–Dindo grade I (OR 1.05; 95% CI 0.72–1.55; *p* = 0.79), Clavien–Dindo grade II (OR 0.47; 95% CI 0.23–0.98; *p* = 0.04), Clavien–Dindo grades III–V (OR 1.11; 95% CI 0.52–2.36; *p* = 0.79; Table 2).

## Discussion

The f-URS has been used in the field of urology for more than 40 years and the development of f-URS is perfectly in accordance with the concept of urology in the field of minimally invasive surgery. In recent decades, the studies reported on f-URS have increased (23). A meta-analysis has shown that, compared with extracorporeal shock wave lithotripsy (ESWL), f-URS could successfully treat the patients with stones <2 cm, with a higher SFR, especially 1–2 cm in the lower pole (24).

However, the limitations in conventional f-URS, i.e., ru-fURS, contain a high initial purchase cost, expensive repair, a risk of cross-infection, and durability at a later stage. To overcome some limitations of ru-fURS, the conception of su-fURS has been proposed (25). It is, particularly, important to discuss the difference between the su-fURS and ru-fURS. As far as we know, this is the first meta-analysis to explore the differences between the two f-URS that aimed to provide medical evidence for clinicians to choose the appropriate approach.

**Figure 3:**
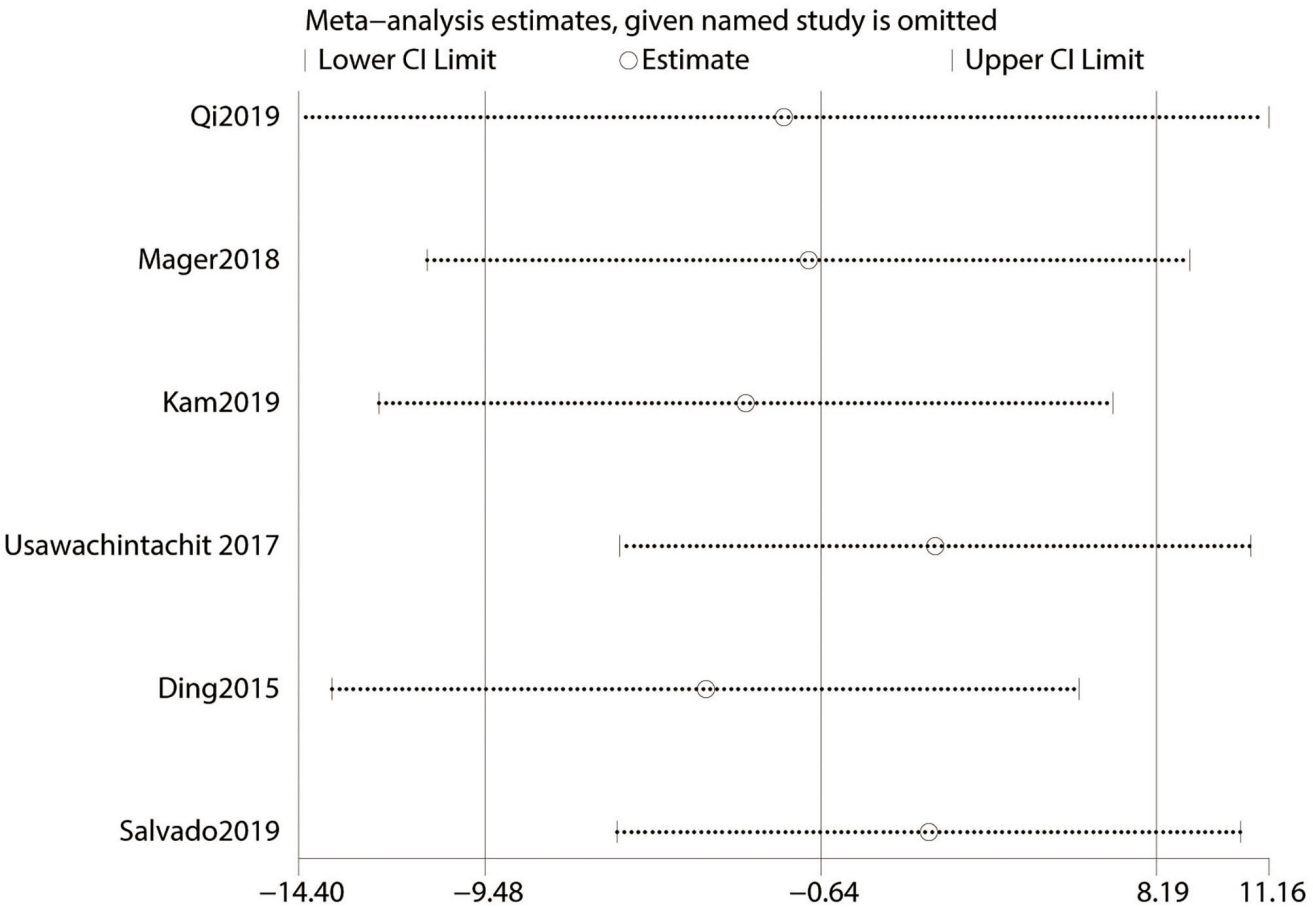
Sensitivity analysis of operative time.

After a sensitivity analysis (Figure 3), this meta-analysis of OT was eventually included in six studies. The final result indicated that no significant differences exist between the two f-URS. It could be seen that the OT of su-fURS procedure was more than ru-fURS in Ding et al. study (16). Professor Ding et al. used a kind of su-fURS, which was named Polyscope and developed in 2011. The surgeon adjusted the degree of deflection of the Polyscope by the force to squeeze the handle constantly and become fatigued during prolonged operation, especially for stones in the lower calyces (16). In addition, the Polyscope lacked two-way deflection, which could not only increase the difficulty in operation but also cause, sometimes, loss of navigation control (26). These causes all bring out increased OT of su-fURS. On the contrary, Salvado et al. reported the use of su-fURS that was the Uscope (PU3022), which was developed in 2017. It has a special self-locking technology that could reduce the fatigue of the surgeon (27). We speculate that the difference between su-fURS themselves was the main reason. Different definitions of time and technical proficiency of the surgeon are also important factors affecting the time of OT.

In terms of EBL, LOS, and SFR, our meta-analysis showed that no significant statistical difference. Since the introduction of su-fURS, it has undergone an evaluation of a series of *in vitro* experiments. Several studies have indicated that there were no remarkable differences in image quality and deflection ability between the su-fURS and ru-fURS (28, 29). Different stone locations, calyceal structures, long learning curves, and surgeon proficiency are all probable reasons.

The previous study (30), which compared the su-fURS and ru-fURS for the renal stones, was different from our study results, especially in terms of OT and SFR. Sometimes, the SDs are not presented in the article and researchers need to estimate SDs from other related information such as standard errors, confidence intervals, *p*-values, and *t*-values. Different calculation methods may be the reasons. In addition, the sensitivity analysis results of their study are not stable, which means that results were greatly affected by bias and should be very careful when concluding results.

Our final meta-analysis results about complications and demonstrates that no statistical difference exists between the su-fURS and ru-fURS. In addition, we did subgroup analysis according to the Clavien–Dindo grades, and the statistical difference can only be found in the Clavien–Dindo grade II (OR 0.47; 95% CI 0.23–0.98; *p* = 0.04; Table 2). According to the existing studies, most complications were grade I–III (98%) and the most common complication in grade II is urinary tract infection (31). Studies have shown that even when ru-fURS was cleaned manually and disinfected by hydrogen peroxide gas, contamination could still be found (7, 8). This may lead to cross-infection between the patients (32). The amount of cleaning and disinfection could also affect the service life of the extremely fragile equipment. On the other hand, the su-fURS automatically eliminates the risk of contamination (25). However, many factors could affect the occurrence of postoperative infections and the definition of urinary tract infection also was the influencing factor. Generally speaking, the outcome needs to be with respect to caution.

The greatest advantage of su-fURS is its cost-effectiveness, which means lower price, no maintenance, and is ready to use. Professor Martin et al. reported a 12-month demographic-based cost-effectiveness analysis in the United States pointing out that the su-fURS and ru-fURS reached the financial breakeven point in 99 cases (33). Another study showed that if the price of su-fURS is no higher than $1,200, it would be more economical (9). One study, which included 23 cases (14 cases for URF-P6, nine cases for LithoVue) and was a small sample cost analysis, showed that compared with URF-P6, LithoVue acquisition costs were higher, but savings were achieved in the terms of labor, consumables, and repair. When these factors were taken into account, the total cost of using these two fURS per case was comparable (34). Nevertheless, these studies not only contain the treatment of the upper urinary stones, but also diagnostic and biopsy. The aforementioned research conclusions need to be with respect to caution.

We followed the PRISMA guidelines strictly to perform this meta-analysis (35). However, some limitations cannot be avoided. First, the studies included were not all high-quality RCTs, leading to insufficient levels of evidence. Second, a limited number of clinical studies, so it is not convincing to apply it on a large scale. Third, lacking detailed date, we fail to perform a subgroup analysis of stone location and stone size. Fourth, due to the definition of expenditure and income, we failed to perform a meta-analysis on cost-effectiveness. This was an important inherent limitation of our study.

## Conclusion

In conclusion, our meta-analysis demonstrates that su-fURS, compared with ru-fURS, has similar effectiveness and better security for patients with upper urinary calculi which provides some benefit to medical institutions of less surgical volume. A larger sample size, multicenter, and longer follow-up RCTs are still needed to support our conclusion.

## Data Availability Statement

The original contributions presented in the study are included in the article/Supplementary Material, further inquiries can be directed to the corresponding author/s.

## Author Contributions

YL conceived and designed the experiments. LP and JinzL analyzed the data. JinzL, JinmL, and JW contributed to reagents, materials, and analysis. CM and LP wrote the manuscript. All authors have read and approved the final manuscript.

## Funding

This study was supported by the Promotion Project of Health Suitable Technology of Sichuan Province (18SYJS15) and the Health Commission of Sichuan Province(20PJ305).

## Conflict of Interest

The authors declare that the research was conducted in the absence of any commercial or financial relationships that could be construed as a potential conflict of interest.

## Publisher's Note

All claims expressed in this article are solely those of the authors and do not necessarily represent those of their affiliated organizations, or those of the publisher, the editors and the reviewers. Any product that may be evaluated in this article, or claim that may be made by its manufacturer, is not guaranteed or endorsed by the publisher.
