# Supplementary material for: Comparison Between Single-Use Flexible Ureteroscope and Reusable Flexible Ureteroscope for Upper Urinary Calculi: A Systematic Review and Meta-Analysis
Source: Front Surg. 2021 Oct 13;8:691170. doi: 10.3389/fsurg.2021.691170 (PMC8548426; doi:10.3389/fsurg.2021.691170)
Supplement: Supplementary file 1 [file Table_1.DOCX]

**Table 1** **Baseline characteristic of included studies**

| Studies,  year | Country | Intervention  su-fURS/ru-fURS | No.of  patients | Age  (year) | Number of  stones | Stone size  (mm) | Study  design | Quality  score |
| --- | --- | --- | --- | --- | --- | --- | --- | --- |
| Zhu  (2020) | China | PU3022A  Flex-X2 | 45  45 | 45.1±9.3  44.5±8.5 | NA | 11.6±5.0  8.7±3.0 | RCT | 7^a^ |
| Qi  (2019) | China | ZebraScope  URF-V | 63  63 | 51.84±13.16  53.25±12.11 | 1.17±0.92  1.95±1.02 | NA  NA | RCT | 6^a^ |
| Mager  (2018) | Germany | Lithovue  Flex-X2S, Flex-XC | 60  62 | 54±17  59±16 | NA | NA  NA | prospective | 8^b^ |
| Kam  (2019) | Australia | Lithovue  URF-V2 | 55  64 | 53.5(46.2-60.7)^c^  53.3(47.6-59.0)^c^ | 2.3(1.6-2.9)^c^  2.0(1.7-2.4)^c^ | 14.7(11.2-18.1)^c^  13.3(11.0-15.6)^c^ | prospective | 7^b^ |
| Usawachintachit (2017) | U.S.A | Lithovue  URF-P6 | 92  50 | 55.8±15.1  50.5±12.6 | 2.0±1.7  1.6±1.3 | 14.7±9.9  16.3±12.2 | prospective | 8^b^ |
| Ding  (2015) | China | PolyScope  URF-P5 | 180  180 | 50.5±12.8  51.1±13.7 | 1.53±0.7  1.58±0.94 | NA  NA | RCT | 6^a^ |
| Salvado  (2019) | Chile | Uscope3022  Cobra | 31  30 | 50.4±13.8  49.9±16.5 | NA | 10.8±5.0  9.0±3.3 | prospective | 7^b^ |

su-fURS, single-use flexible ureteroscope; ru-fURS, reusable flexible ureteroscope; NA not available; ±refers to standard deviation;

a using Jadad scale; b using NOS scoring rule; c mean (95%CI).

**Table 2 The meta-analysis of postoperative complication**

| **complication** | **No. of**  **studies** | **No. of patients** | **OR (95% CI)** | **P-Value** | **Heterogeneity(I^2^)** |
| --- | --- | --- | --- | --- | --- |
|  |  | **su-fURS /ru-fURS** |  |  |  |
| Clavien–Dindo grade I | 7 | 526/494 | 1.05(0.72,1.55) | 0.79 | 1.7% |
| Clavien–Dindo grade II | 5 | 315/284 | 0.47(0.23,0.98) | 0.04 | 0% |
| Clavien–Dindo grade III–V | 4 | 395/355 | 1.11(0.52,2.36) | 0.79 | 0% |
| Total | 7 | 526/494 | 0.93(0.66,1.29) | 0.65 | 41.7% |

OR,odds ratios; CI,confidence interval; su-fURS, single-use flexible ureteroscope; ru-fURS, reusable flexible ureteroscope.
