# Supplementary figures and images for: Comparison Between Single-Use Flexible Ureteroscope and Reusable Flexible Ureteroscope for Upper Urinary Calculi: A Systematic Review and Meta-Analysis
Source: Front Surg. 2021 Oct 13;8:691170. doi: 10.3389/fsurg.2021.691170 (PMC8548426; doi:10.3389/fsurg.2021.691170)

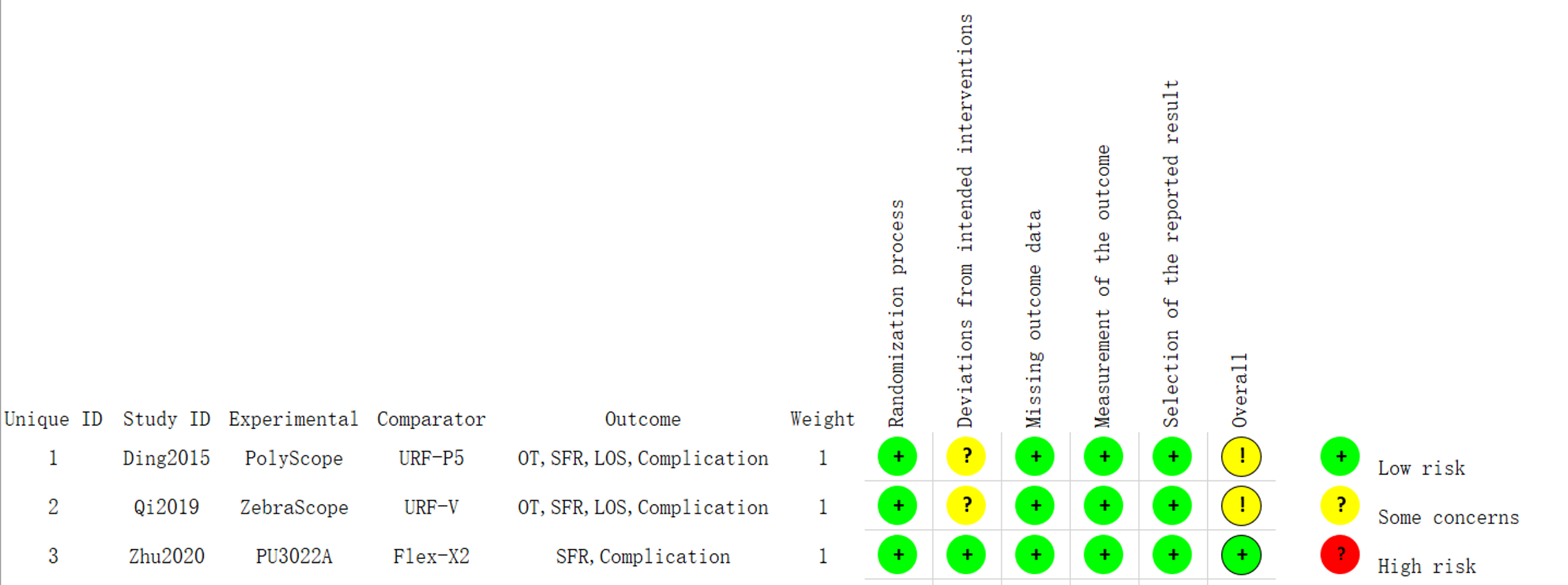

Supplement: Supplementary file 2 [file Image_1.PNG]
